# Determination of Fusobacterium nucleatum levels in patients with periodontal disease and oral squamous cell carcinoma

**DOI:** 10.3205/dgkh000589

**Published:** 2025-09-30

**Authors:** Karthik Shunmugavelu, Bala Geetha Shakthi Chakravarthy, Shanmuga Priya

**Affiliations:** 1 Department of Dentistry, PSP Medical College Hospital and Research Institute Tambaram, Tamil Nadu, India; 2 Department of Community Medicine SRM Medical College Hospital & Research centre, SRM Institute of Science & Technology, Tamil Nadu, India; 3 Anatomy Department, Sree Balaji Medical College and Hospital, Bharath University, Chennai, Tamil Nadu, India

**Keywords:** Fusobacterium nucleatum, periodontal disease, oral squamous cell carcinoma, oral microbiome

## Abstract

**Introduction:**

*Fusobacterium (F.) nucleatum*, a Gram-negative anaerobic bacterium, has been implicated in both periodontal disease and oral squamous cell carcinoma (OSCC). This review aims to evaluate the levels of *F. nucleatum* in patients with periodontal disease and OSCC, exploring its potential role in the pathogenesis.

**Methods:**

A comprehensive literature search was conducted across multiple databases, identifying studies that measured *F. nucleatum* levels in periodontal disease and OSCC tissues.

**Results:**

A higher prevalence of *F. nucleatum* exists in both periodontal disease and OSCC tissues compared to healthy controls.

**Conclusion:**

It appears that there is a link between infection with* F. nucleatum* and the development of these oral diseases. Further research is warranted to elucidate the mechanisms underlying this association and to explore potential therapeutic interventions targeting *F. nucleatum*.

## Zusammenfassung

**Hintergrund:**

*Fusobacterium (F.) nucleatum*, ein Gram-negatives anaerobes Bakterium, wurde sowohl mit Parodontalerkrankungen als auch mit oralen Plattenepithelkarzinomen (OSCC) assoziiert. Ziel der Übersichtsarbeit ist es, die Menge von *F. nucleatum* bei Patienten mit Parodontalerkrankungen und OSCC zu bewerten und die mögliche Rolle bei der Pathogenese dieser Erkrankungen zu untersuchen.

**Methode:**

Es wurde eine umfassende Literaturrecherche in mehreren Datenbanken durchgeführt, um Studien zu identifizieren, in denen die Menge von *F. nucleat*um in Geweben von Parodontalerkrankungen und OSCC bestimmt wurde.

**Ergebnisse:**

Die Ergebnisse weisen auf eine höhere Prävalenz von *F. nucleatum* sowohl in parodontal erkranktem als auch in OSCC-Gewebe im Vergleich zu gesunden Kontrollen hin.

**Schlussfolgerung:**

Offenbar besteht ein Zusammenhang zwischen einer Infektion mit *F. nuc*leatum und der Entwicklung dieser oralen Erkrankungen. Weitere Forschungsarbeiten sind erforderlich, um die diesem Zusammenhang zugrunde liegenden Mechanismen aufzuklären und mögliche therapeutische Maßnahmen gegen *F. nucleatum* zu untersuchen.

## Introduction

Periodontal diseases encompass inflammatory conditions affecting the supporting structures of the teeth, primarily caused by bacterial infections. *Fusobacterium (F.) nucleatum*, a key periodontal pathogen, has been frequently isolated from periodontal pockets and is known to play a significant role in the progression of periodontal disease. Recent studies have also identified *F. nucleatum* in various malignancies, including colorectal cancer and oral squamous cell carcinoma (OSCC), suggesting a potential oncogenic role. The review aims to assess the levels of *F. nucleatum* in patients with periodontal diseases and OSCC, providing insights into its potential involvement in the pathogenesis of these conditions [1], [2].

## Methods

A systematic literature search was conducted using databases such as PubMed, Scopus, Web of Science, Embase, and Cochrane, covering studies published up to March 2025. The search strategy used the keywords “*Fusobacterium* *nucleatum*”, “periodontal disease”, “oral squamous cell carcinoma”, and “oral microbiome”. Inclusion criteria encompassed studies that quantitatively measured *F. nucleatum* levels in periodontal disease or OSCC tissues. Exclusion criteria involved studies lacking quantitative data or focusing on other *Fusobacterium* spp. Data extraction was performed independently by two reviewers, and discrepancies were resolved through discussion [3].

## Results

The initial search yielded 118 unique records, with 88 full-text articles assessed for eligibility. Seventeen (17) studies met the inclusion criteria, of which we reviewed 5 (Table 1 (Tab. 1)). Although 18 studies initially met the inclusion criteria, we included only 5 in the final review to ensure methodological consistency and data integrity. The excluded studies exhibited significant limitations, including inadequate reporting of quantitative outcomes, inconsistent microbial detection methods (e.g., qPCR vs. 16S rRNA sequencing), and population variability that introduced substantial heterogeneity. Additionally, several studies were assessed as having a high risk of bias, further reducing their suitability for inclusion. By focusing on a smaller subset of high-quality studies, we aimed to enhance the reliability of our findings and align with PRISMA recommendations for transparent and reproducible systematic reviews.

In periodontal diseases, *F. nucleatum* was more abundant than in healthy controls, with detection rates ranging from 57.1% to 68% in patients with gingivitis and periodontitis, respectively, and 37.8% in healthy individuals. In OSCC tissues, *F. nucleatum* was present in 16% of tumor lesions compared to 10% in non-tumor lesions, indicating a higher prevalence in cancerous tissues. A pilot case-control study reported a 25% detection rate of *F. nucleatum* in OSCC tissues, while it was not present in any of the control samples.

## Discussion

*F. nucleatum* is a highly adaptable and opportunistic bacterium widely present in the human oral microbiome. The physiological and metabolic versatility of this organism has been demonstrated in various studies, highlighting its role in different pathological conditions, including periodontal diseases, OSCC, and systemic diseases [4].

The study by Rogers [5] explored the metabolic abilities of *F. nucleatum*, showing that it can ferment both simple carbohydrates (such as glucose and fructose) and amino acids, either free or in small peptides. This metabolic flexibility allows it to survive in various niches within the oral cavity, including supra- and sub-gingival dental plaque. Aminopeptidase activity was found to be essential for growth in peptide-rich environments, suggesting that this enzymatic function plays a key role in its ecological adaptability. Additionally, the study raised doubts about the validity of the current subspecies classification of *F. nucleatum* based on allozyme electrophoresis. Although its presence in periodontal disease-associated bacterial consortia is well-documented, its exact contribution to disease progression remains uncertain.

Chen et al. [1] provided a comprehensive overview of *F. nucleatum* as more than just a periodontal pathogen, highlighting its association with various oral and systemic diseases. The study emphasized that *F. nucleatum* is enriched in conditions such as halitosis, dental pulp infections, and oral cancer and contributes to inflammation, immune modulation, and potentially tumor progression. The authors discussed emerging therapeutic strategies targeting *F. nucleatum* and the importance of understanding its pathogenic mechanisms to develop novel diagnostic and therapeutic interventions.

Kaliamoorthy et al. [2] conducted a pilot case-control study investigating the presence of *F. nucleatum* in OSCC tissue samples. The study detected the bacterium in 25% of OSCC cases, whereas it was absent in non-cancerous controls, reinforcing the hypothesis that *F. nucleatum* may be involved in oral carcinogenesis. The study suggested its potential use as a biomarker for early diagnosis, risk assessment, and prognosis of OSCC. However, given the limited sample size, the authors emphasized the need for larger-scale studies to confirm these findings and elucidate the underlying mechanisms of its role in cancer progression.

A meta-analysis by Bronzato et al. [3] further supported the link between *Fusobacterium* and cancer, demonstrating a significantly higher prevalence of *Fusobacterium* spp. in tumor lesions compared to non-tumor lesions. *F. nucleatum* was identified as the most prevalent species, detected in nearly 47.06% of cases. The study also highlighted that molecular-based methods were the most reliable for detecting *Fusobacterium*, accounting for 64.7% of positive cases. These findings suggest a possible role of *Fusobacterium* in the development of oral and head and neck cancers, warranting further research to determine its precise contribution to tumorigenesis.

Neuzillet et al. [6] provided additional insights into the prognostic value of intratumoral *F. nucleatum* and its association with immune-related gene expression in OSCC patients. The study analyzed a large cohort of 151 OSCC patients and found that *F. nucleatum* positivity was associated with a favorable prognosis, including longer overall survival, relapse-free survival, and metastasis-free survival. Additionally, the study revealed a distinct immune microenvironment in *F. nucleatum* positive OSCC cases, where a higher Gram-negative bacterial load correlated inversely with M2 macrophage presence. These findings suggest that *F. nucleatum* may influence tumor immunity, which could have implications for immunotherapeutic strategies in OSCC management.

The findings from these studies collectively underscore the complex role of *F. nucleatum* in oral health and disease. While its metabolic adaptability allows it to colonize diverse environments, its potential pathogenicity in both periodontal and systemic diseases, particularly OSCC, remains an area of active investigation. The emerging evidence linking *F. nucleatum* to cancer progression and immune modulation highlights the need for further research to determine whether this bacterium serves as a causative agent or merely an opportunistic colonizer. Understanding its interactions with host immunity and other microbial species may open new avenues for targeted therapeutic interventions and improve diagnostic accuracy in oral and systemic diseases.

## Conclusion

*F. nucleatum* is a metabolically versatile bacterium that plays a significant role in both oral and systemic diseases. Its ability to ferment carbohydrates and amino acids allows it to thrive in diverse oral environments, contributing to its presence in both supra- and sub-gingival dental plaque. While traditionally associated with periodontal disease, emerging research highlights its involvement in various pathological conditions, including oral squamous cell carcinoma (OSCC) and other head and neck cancers. Studies have demonstrated its increased prevalence in tumor tissues and its potential as a biomarker for cancer diagnosis and prognosis. Moreover, its impact on immune modulation suggests a possible role in influencing disease progression.

Despite these insights, the exact contribution of *F. nucleatum* to disease remains unclear. While some studies link its presence to pathogenic outcomes, others suggest a potential association with better survival rates in certain cancers. This paradox underlines the need for further research to delineate its mechanisms of action, interactions within microbial consortia, and potential therapeutic interventions. Understanding the metabolic and physiological characteristics of* F. nucleatum* could pave the way for new diagnostic and treatment strategies, ultimately improving patient outcomes in both oral and systemic health.

## Notes

### Competing interests

The authors declare that they have no competing inter-ests.

### Funding

None. 

### Authors’ ORCIDs

- Shunmugavelu K: https://orcid.org/0000-0001-7562-8802
- Shakthi Chakravarthy BG: https://orcid.org/0009-0006-0582-1563

## Figures and Tables

**Table 1:**
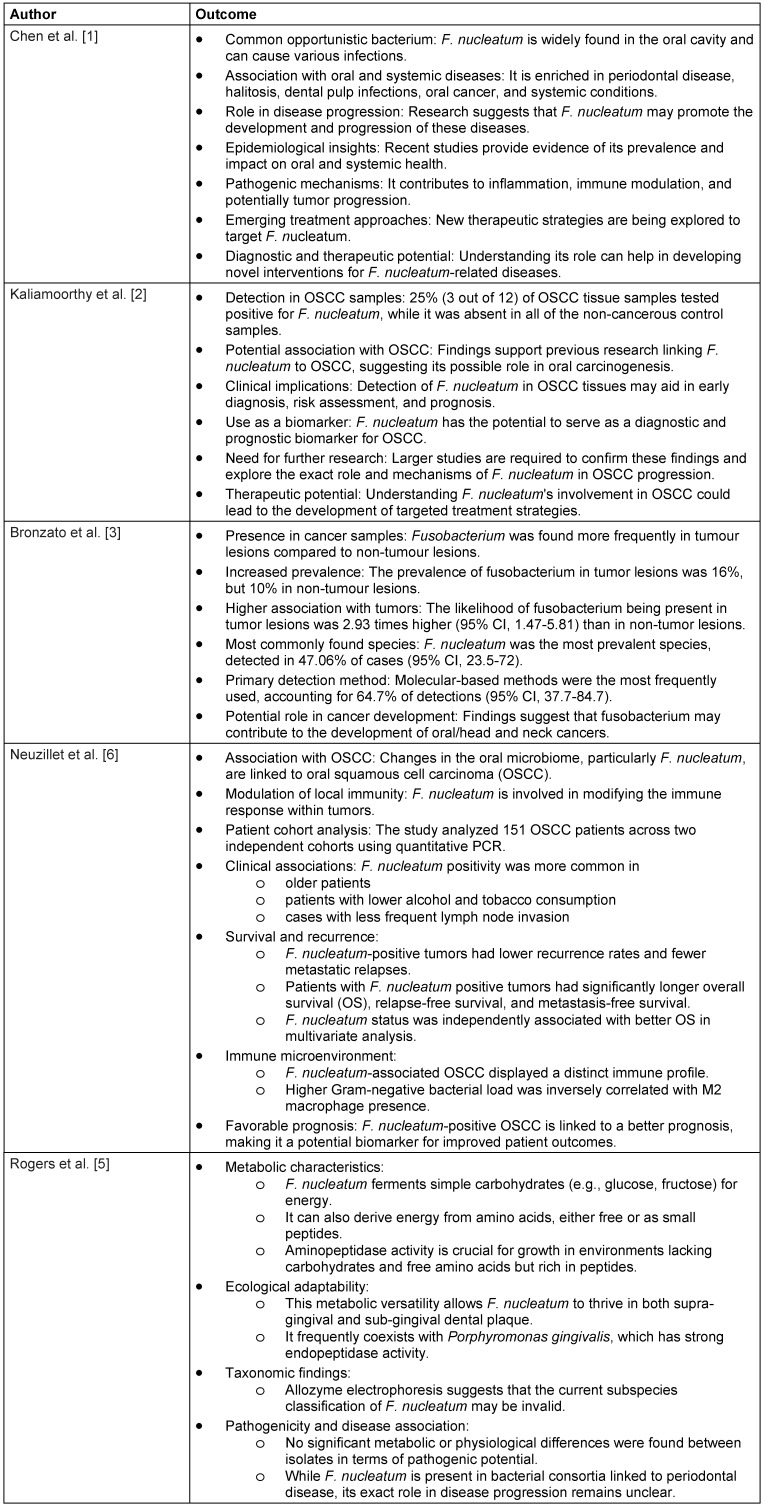
Key outcomes of the five analyzed studies

## References

[1] Chen Y, Huang Z, Tang Z, Huang Y, Huang M, Liu H, Ziebolz D, Schmalz G, Jia B, Zhao J (2022). More Than Just a Periodontal Pathogen -the Research Progress on Fusobacterium nucleatum. Front Cell Infect Microbiol.

[2] Kaliamoorthy S, Priya Sayeeram S, SundarRaj S, Balakrishnan J, Nagarajan M, Samidorai A (2023). Investigating the Association Between Fusobacterium nucleatum and Oral Squamous Cell Carcinoma: A Pilot Case-Control Study on Tissue Samples. Cureus.

[3] Bronzato JD, Bomfim RA, Edwards DH, Crouch D, Hector MP, Gomes BPFA (2020). Detection of Fusobacterium in oral and head and neck cancer samples: A systematic review and meta-analysis. Arch Oral Biol.

[4] McIlvanna E, Linden GJ, Craig SG, Lundy FT, James JA (2021). Fusobacterium nucleatum and oral cancer: a critical review. BMC Cancer.

[5] Rogers AH (1998). Studies on fusobacteria associated with periodontal diseases. Aust Dent J.

[6] Neuzillet C, Marchais M, Vacher S, Hilmi M, Schnitzler A, Meseure D, Leclere R, Lecerf C, Dubot C, Jeannot E, Klijanienko J, Mariani O, Calugaru V, Hoffmann C, Lesnik M, Badois N, Borcoman E, Piaggio E, Kamal M, Le Tourneau C, Bieche I (2021). Prognostic value of intratumoral Fusobacterium nucleatum and association with immune-related gene expression in oral squamous cell carcinoma patients. Sci Rep.

